# Spatial Eosinophil Phenotypes as Immunopathogenic Determinants in Inflammatory Diseases

**DOI:** 10.3390/cells14110847

**Published:** 2025-06-05

**Authors:** Jonas S. Erjefält

**Affiliations:** 1 Unit of Airway Inflammation, Department of Experimental Medical Science, Lund University, 221 84 Lund, Sweden; jonas.erjefalt@med.lu.se; 2 Department of Respiratory Medicine and Allergology, Lund University, 221 85 Lund, Sweden

**Keywords:** eosinophils, type 2 immunity, degranulation, immunopathology

## Abstract

Eosinophils are increasingly recognized as adaptable immune cells that exhibit diverse phenotypes and effector functions across different tissues and disease states. While they can induce pathology through degranulation and cytotoxic mediator release, eosinophils also fulfill regulatory and tissue repair roles. Advances in single-cell and spatial technologies have begun to reveal how microenvironmental cues (including cytokines, chemokines, and cell–cell interactions) shape eosinophil behavior in health and disease. These insights are critical for understanding why certain patients respond variably to therapies targeting eosinophils and related type 2 pathways. By dissecting eosinophil heterogeneity in real human tissues, researchers may identify new biomarkers, refine endotyping approaches, and develop more precise therapeutic strategies. This review summarizes emerging concepts of eosinophil biology in inflammatory conditions, highlights the impact of spatial context on eosinophil functions, and discusses the future of advanced phenotyping in guiding personalized treatments.

## 1. Introduction

Eosinophils are granulocytic leukocytes traditionally associated with helminth infections and atopic disease states, notably in allergic asthma, atopic dermatitis (AD), and eosinophilic esophagitis (EoE) [1,2,3]. In healthy individuals, eosinophils reside primarily in the gastrointestinal tract and to some extent in other tissues, where they are thought to fulfill homeostatic roles, including tissue remodeling and repair [4,5]. However, in a range of inflammatory diseases, eosinophil levels in blood and diseased tissue compartments may increase dramatically. It is generally believed that such an accumulation is pathogenic and associated with the activation of type 2 inflammatory pathways [6,7,8].

While the classical narrative portrays eosinophils as potent effector cells causing tissue damage and inflammation via release of cytotoxic granule proteins, emerging data suggest a far more nuanced picture. Recent studies indicate that eosinophils possess sophisticated immunomodulatory functions, can promote tissue repair, and adopt distinct phenotypes depending on spatial microenvironmental contexts [4,9]. The concept of eosinophil heterogeneity, whereby distinct subsets or states co-exist and serve different pathological or homeostatic functions, has gained traction in recent years [3,10,11,12,13]. Further, microenvironments with eosinophilic inflammation may occur alongside spatially distinct regions with a non-eosinophilic type of inflammation [14]. Understanding this complexity in real human tissues is crucial for improved understanding and treatment of eosinophil-associated diseases. Such knowledge may also explain why biologic treatments targeting eosinophils have different efficacy across patients or diseases with similar degrees of eosinophilia.

This review focuses on the emerging picture of eosinophil heterogeneity and effector functions in diseased human tissues. It also discusses the evolving understanding of how eosinophils contribute to both immunopathology and homeostasis, the still enigmatic role of type 2 inflammation and IL-5–independent pathways, and the impact of anatomical localization. Also considered is how advanced phenotyping and novel spatially resolved single-cell technologies will help researchers to decode disease-relevant eosinophil activities within patient tissues. Finally, we will highlight the implications of these insights for the pharmacological targeting of eosinophils and improving clinical outcomes.

## 2. Factors Governing Eosinophil Maturation, Tissue Recruitment, and Activation

Eosinophil development and recruitment are orchestrated by a finely tuned network of cytokines and chemokines. Interleukin 5 (IL-5) is the central driver of eosinophilopoiesis and maturation in the bone marrow [15,16]. In eosinophil conditions, increased IL-5 signaling leads to the expansion of the eosinophil progenitor pool and the subsequent release of mature eosinophils into the bloodstream. Chemokines, particularly the eotaxin family (CCL11, CCL24, and CCL26), guide eosinophils from circulation into tissues, where local signals further shape their activation state [2,3].

**Figure 1:**
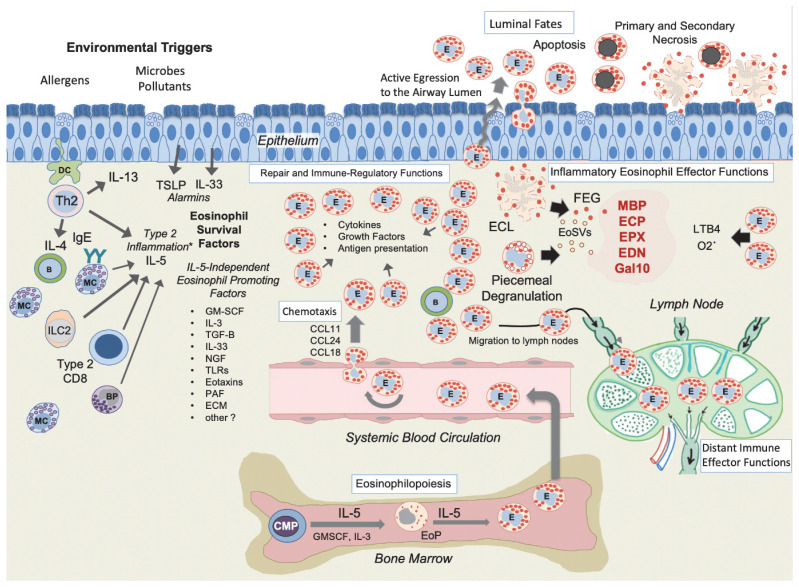
A schematic overview of eosinophil maturation, tissue homing, effector function modes, and fates of airway mucosal eosinophils (white boxes). Shown are also routes for the active egression of tissue eosinophils into the airway lumen or to lung-draining lymph nodes, as well as common fates of luminal eosinophils. To the left are also factors promoting longevity and survival of tissue eosinophils. Whereas the eosinophilopoiesis in the bone marrow and establishment of a circulating eosinophil pool is highly IL-5 dependent, the relative proportion of IL-5 versus IL-5-independent factors for maintaining the tissue eosinophila is dynamic and highly context-dependent. * Type 2 inflammation, as depicted here, is a simplified representation; its execution in actual patient tissues remains largely unknown. Abbreviations: ECL = eosinophil cytolysis; FEG = free eosinophil granules; EoSVs = eosinophil sombrero vesicles; Th2 = T helper type 2 cells (GATA3+ CD4 cells); ILC2 = type 2 innate lymphoid cells; BP = basophil; DC = dendritic cell.

Tissue eosinophilia is thought to be intimately linked with type 2 (T2) inflammation, a response characterized by the production of IL-4, IL-5, and IL-13 [8,17,18]. The type 2 cytokine response may in turn be caused by underlying sensitization and early allergen-evoked immune responses, or by upstream acute immune responses effectuated by release of alarmins such as IL-33 and TSLP [19] (Figure 1).

Classical type 2 inflammation has been viewed as the underlying immunological driver in eosinophilic conditions like allergic asthma, allergic rhinitis, atopic dermatitis, and EoE [8,17,20,21,22,23]. However, recent findings suggest type 2 inflammation in most eosinophilic conditions to be surprisingly multifaceted and complex, with many context-dependent variants in terms of induction and execution modes [19,24,25]. The heterogeneity concerns both the proportions and cellular sources of type 2 cytokines. Indeed, not only classical CD4 Th2 cells and ILC2s, which are normally forwarded as orchestrators and producers of the type 2 cytokines, but other cell types including type 2-biased CD8^+^ T cells, basophils, and mast cells, may also contribute [26,27,28,29]. Importantly, although many cells in tissue areas with eosinophilia are T2-capable, only a limited subfraction are actively secreting effector cytokines. Which of the cell types that execute functional type 2 responses is currently under intense investigation across several eosinophilic diseases.

Whereas IL-5 is critical for eosinophilopoiesis in the bone marrow, it is increasingly recognized that the maintenance of an established tissue eosinophilia may occur independently of IL-5-driven local tissue type 2 signals [18,30,31]. This phenomenon may help explain why some patients respond poorly to anti-IL-5/IL-5Ra therapies despite having elevated eosinophil counts. Better understanding of pathways promoting the activation and survival of tissue eosinophils is critical, as they may influence the success of eosinophil-targeted biologics. In any case, the heterogeneity in clinical response across patients with tissue eosinophilia challenges the simplistic view that all eosinophil-rich diseases are uniformly type 2-driven and underscores the need for more nuanced endotyping.

## 3. Eosinophil Effector Functions in Diseased Tissues: Beyond Granule Protein Release

Traditionally, eosinophils are best known for their cytotoxic armamentarium, anchored by the release of highly basic and cationic proteins contained within secondary granules. The four major eosinophil granule proteins, major basic protein (MBP), eosinophil cationic protein (ECP), eosinophil peroxidase (EPX), and eosinophil-derived neurotoxin (EDN), have historically defined eosinophil effector function [3,9,32]. These proteins can cause epithelial damage, neuronal dysfunctions, bronchoconstriction, and mucus hypersecretion [2,32,33,34,35], and their presence in tissues and biofluids is associated with disease severity in eosinophilic disorders. Elevated levels of MBP and ECP, for example, can be detected in the bronchoalveolar lavage (BAL) fluid, sputum, and blood of patients with severe eosinophilic asthma, correlating with airflow obstruction and exacerbation frequency [32,35,36].

Eosinophils employ distinct modes of degranulation. Piecemeal degranulation (PMD) is a selective and controlled mechanism where granule contents are slowly mobilized and released, enabling fine-tuned effects [9,37,38]. During classical or compound exocytosis, entire granule packages are released from living cells via plasma membrane fusion [39]. In contrast, eosinophil cytolysis (ECL) involves the release of intact granules via a programmed non-apoptotic cell death [34,37]. Evidence for eosinophil degranulation and ECL in human disease has been derived from ultrastructural analyses of tissue biopsies, immunostaining for extracellular granule proteins, and the detection of soluble granule proteins in various clinical samples [37,40,41]. Whereas in patients, PMD and ECL together constitute the bulk of granule protein release, ECL evokes inflammatory signals also through chromatolysis and the formation of extracellular dsDNA traps and pro-inflammatory galactin-10 containing Charcot–Leyden crystals [35,41,42,43,44,45].

Despite the broad pathogenic potential of ECL and PMD, their molecular triggers and regulation remain relatively unknown. However, recent data suggest that each degranulation type has distinct receptor molecular triggers [38]. Also, the mechanistic execution is different with intricate mechanisms for the selective and controlled release of granule content [41,46]. For example, the extracellular membrane-bound granules liberated by ECL act like independent immune responders and have surface cytokine and leukotriene receptors controlling the subsequent content release [41,46]. New data have also revealed that during both PMD and ECL granules release eosinophil sombrero vesicles (EoSVs) for selective protein release and, potentially, direct communication to neighboring immune cells [41,44,47].

However, bulk mediator release through degranulation is not the whole story. Eosinophils can also perform a fine-tuned release of an array of cytokines (e.g., IL-4, IL-13, TGF-β), chemokines (e.g., CCL11/eotaxin), lipid mediators (e.g., leukotrienes and prostaglandins), and growth factors (e.g., vascular endothelial growth factor) [11,12,48,49]. These mediators enable eosinophils to influence neighboring immune cells, shaping T helper 2 (Th2) responses, interacting with group 2 innate lymphoid cells (ILC2s), and modulating dendritic cell function [4,13,50,51,52,53,54]. There is also significant evidence of eosinophils acting as antigen-presenting cells (see section on anatomical compartment below). Apart from degranulation and immunomodulation, eosinophils can also participate in tissue remodeling and repair processes, stimulating fibroblasts, epithelial cells, and smooth muscle cells to produce extracellular matrix components [13,18,48]. Thus, eosinophils can adopt both tissue-damaging and tissue-protective roles, and it is likely that in many clinical conditions, both pathogenic pro-inflammatory and tissue-protecting mechanisms occur in parallel. Naturally, this double-edged feature of eosinophils is important to consider when evaluating the role of eosinophils in specific clinical settings.

## 4. Heterogeneity and Fates of Eosinophils in Human Disease

Early studies distinguished “normodense” from “hyperdense” eosinophils based on gradient density separation, suggesting the existence of distinct eosinophil subpopulations [55]. More recent conceptual frameworks propose that eosinophils can be categorized broadly into “homeostatic” and “inflammatory” subsets [10,56]. Homeostatic eosinophils are proposed to reside in in tissues under steady-state conditions, supporting tissue maintenance and integrity, whereas inflammatory (or “inducible”) eosinophils are rapidly recruited during disease flares, are highly activated, and produce more pro-inflammatory mediators [10,55,56]. While being conceptually attractive, this binary classification is however an oversimplification. For instance, single-cell RNA sequencing (scRNA-seq) and mass cytometry have begun to uncover other eosinophil subpopulations with other “distinct” transcriptomic and surface marker profiles [4,48,57]. These approaches have revealed that eosinophil phenotypes vary depending on disease severity, anatomical location, and exposure to local tissue cytokines and chemokines. Although many of these high-dimensional data sets have been generated from animal models or from in vitro-differentiated eosinophils, emerging human data suggest considerable eosinophil diversity also in diseases such as asthma, chronic rhinosinusitis, and EoE [58,59,60].

In summary, though the characterization of eosinophil “subsets” remains in its infancy, it seems clear that their identities and roles in human disease are highly plastic and context-dependent. This is supported by the typical patchy spatial distribution patterns of eosinophils and varying microenvironmental composition of eosinophil-modulating factors (IL-33, TSLP, PAF, IL-5, eotaxins, etc.) across tissue microenvironments [52,61,62,63]. Thus, whereas it seems too simplistic to categorize eosinophils into fixed stereotypic subtypes, eosinophils are a vastly heterogeneous population in inflamed tissues with some phenotypes or effector functions driving pathology and others exerting protective or regulatory effects. Unraveling this complexity in human disease settings is a key challenge for future research.

**Figure 2:**
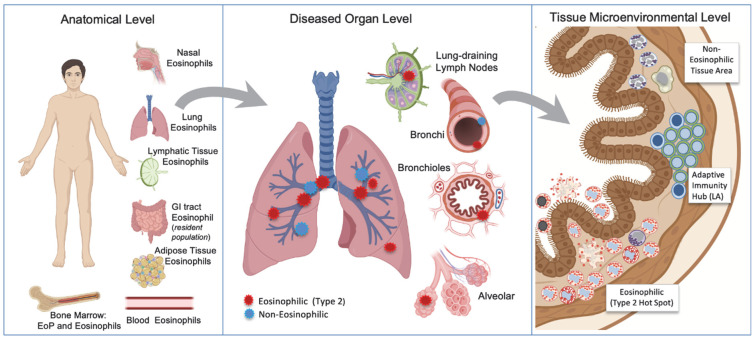
A schematic overview of the spatial distribution levels of eosinophils in asthma and COPD patients classified as “type 2 patients” based on elevated blood (or luminal) eosinophils. Apart from the expected eosinophil infiltration in the lung, eosinophils may variably also be present in the nasal mucosa (especially in atopic asthmatics with CRSwNP), lymph nodes [70], or adipose tissue [76]. In common to healthy individuals, asthma and COPD patients also have a gastrointestinal (GI) population of resident eosinophils. Not only in COPD, but also in asthma the lung eosinophil tissue infiltration may variably also involve the small airways (bronchioles), or even the alveolar parenchyma [65,77]. The tissue eosinophilia is typically patchy [61], both at an anatomical (middle panel) and microenvironmental level (right panel). Importantly, both at an anatomical and microenvironmental level, spatially distinct “hot spots” with aggravated eosinophilia may occur alongside tissue areas subjected to a non-eosinophilic inflammation, creating a mixed-inflammatory phenotype that complicate the anti-inflammatory treatment [14]. EoP = eosinophil progenitor: LA = lymphoid aggregate. CRSwNP = chronic rhinosinusitis with nasal polyps.

The presence and tissue distribution of eosinophils is in most eosinophilic conditions spatially multifaceted and more complex than a mere presence in the main target organ (exemplified in Figure 2). Where eosinophils localize within tissues may be as important as how many are present. In asthma, for example, some patients harbor a more “distal” type 2 inflammation, with eosinophils and Th2 cells accumulating in the small airways or even in the alveolar parenchyma [63,64,65,66,67]. Inhaled corticosteroids may be less effective in such patients, as drug deposition in the peripheral lung is suboptimal [65]. Similarly, the distribution of eosinophils within the airway wall—e.g., within smooth muscle bundles, close to nerves, or in perivascular regions—may influence airway hyperresponsiveness (AHR) and treatment responses [33].

In addition to their presence at the lumen–tissue interface, eosinophils can also migrate to draining lymph nodes [68,69,70] (Figure 1 and Figure 2), where they may shape adaptive immune responses via, e.g., antigen presentation. Indeed, eosinophils have been reported to express key molecules characteristic to professional antigen-presenting cells like HLA-DR/MHC-II, CD80, CD86, and CCRR7 [68,69,71,72]. Moreover, inside lymph nodes, eosinophils may regulate T cell proliferation and influence class switching in B cells. Although the importance of lymph node-resident eosinophils in human disease remains underexplored, it represents a fertile area of investigation. Lymph nodes as an anatomical arena for eosinophil immunopathological activities constitutes a challenge from a drug delivery perspective, especially in respiratory diseases since inhalation therapy does not reach the lung-draining lymph nodes. For eosinophils in hollow organs, another anatomical re-localization mode is active egression into the lumen. At least in respiratory eosinophilic conditions, this represents a bulk fate of tissue eosinophils that can function both as a first line defense and a physiological clearance of eosinophils (Figure 1) [73,74,75]. Naturally, the dramatical alteration of the physical and molecular milieu in the lumen will affect the eosinophil phenotype [74], a fact important to consider when evaluating BAL or sputum eosinophils.

In addition to the programmed process of eosinophil cytolysis (ECL), eosinophils can also undergo primary and secondary necrosis. Primary necrosis involves the loss of plasma membrane integrity due to “accidental” cell injury, causing the release of pro-inflammatory intracellular content. Secondary necrosis occurs when apoptotic eosinophils are not promptly removed by macrophage phagocytosis or mucociliary clearance, eventually leading to membrane rupture but resulting in reduced inflammatory consequences compared to primary necrosis. One pathogenic implication of tethered luminal plugs, which trap cells and prevent their physiological clearance by the mucociliary escalator, could be to increase the necrosis rate of eosinophils.

## 5. Pharmacological Targeting of Eosinophils

Therapeutic strategies for eosinophilic disorders historically relied on glucocorticoids to suppress eosinophilia and broadly dampen inflammation [23,78,79]. Systemic and inhaled corticosteroids remain mainstays of treatment for eosinophilic diseases, with a main proposed mechanism of action being T2 suppression and reduction in eosinophil-promoting IL-5 and eotaxin production. However, steroids have significant side effects and may be insufficient to control severe disease.

The development of biologic therapies directly targeting eosinophils or the pathways governing their survival and recruitment represents a major therapeutic advance. Monoclonal antibodies targeting IL-5 (e.g., mepolizumab, reslizumab) or the IL-5 receptor alpha chain (benralizumab) effectively reduce eosinophil counts in blood and tissues, improving outcomes in many patients with severe eosinophilic asthma [3,80,81,82].

Another anti-eosinophil target is Siglec-8, an inhibitory receptor expressed on eosinophils and mast cells. The investigational drug and siglec-8–agonist antibody lirentelimab induced eosinophil apoptosis [83] and thereby robustly reduces both blood and tissue eosinophil numbers [83]. However, after clinical trials in EGIDs, AD, and urticaria, the clinical efficacy has so far been disappointing. It is of note that the lack of clinical improvement in eosinophilic duodenitis (EoD) was observed despite a successful reduction in tissue eosinophils, highlighting the general difficulties to judge the relative importance of eosinophils to the overall pathogenesis in eosinophilic diseases.

Identifying the right patients for eosinophil-targeted therapies has largely relied on measuring blood eosinophil counts [84,85]. Yet, these approaches have limitations. Elevated blood eosinophils do not always predict tissue eosinophil levels, activity, or response to therapy [40,86,87]. Further, tissue eosinophilia per se may not even predict response to eosinophil-targeted treatments, as illustrated in EoE where eosinophil targeting by benralizumab (anti-IL5Ra) or Siglec-8 activation has shown limited clinical efficacy despite clear proven tissue eosinophilia prior to biologic treatment and successful eosinophil reduction [88,89]. Similarly, while anti-IL-5/IL-5Ra therapies are effective in a large proportion of eosinophilic severe asthmatics, some patients respond poorly despite displaying an eosinophil signature [90,91,92]. Several factors could explain this discrepancy, including the presence of parallel non-type 2 inflammatory pathways [14], genetic type 2 variants [93], alternative eosinophil survival signals in the tissue [31,94], or treatment-resistant eosinophil subsets at distinct anatomical sites [48,63]. In this regard, it is important to not only view eosinophil numbers per se as a predictor of clinical response but also degranulation status. Indeed, eosinophil conditions with similar degrees of tissue eosinophils may display markedly different levels of degranulation [40]. The rational for assessing activity rather that cell density is further underscored by sputum studies [95], and even using ECL-evoked free granules in sputum to guide response to biologic treatment [96]. In further support of this theme, eosinophil peroxidase was recently forwarded as a more reliable biomarker of active eosinophil inflammation than mere cell numbers [97].

Like there are different reasons for lack of clinical efficacy with eosinophil-targeting therapies despite elevated eosinophil counts, the mechanism of action among patients that do respond could also be multifaceted. Although the IL-5/IL-5Ra biologics are commonly forwarded as anti-eosinophil therapies, cells other than eosinophils may express IL-5Ra. Apart from basophils, B cells/plasmablasts as well as the airway epithelium and fibroblasts may variably express IL-5Ra [98,99,100,101]. Similarly, the effects of Siglec-8 activation by lirentimab may not be unique to eosinophils since mast cells and, to a lesser degree, basophils also express Siglec-8 [102,103]. Also, to what extent direct effects on IL5Ra-positive non-eosinophil cell types contribute to clinical efficacy in responders to benralizumab, reslizumab, and mepolizumab remains to be explored.

To complement the directly eosinophil-targeting biologics, there are several therapeutic approaches that indirectly modulate eosinophilic inflammation by altering T2 inflammation or its upstream alarmin pathways. Among these are dupilumab that targets IL-4Rα (dupilumab), thereby blocking IL-4 and IL-13 signaling, or the anti-TSLP MoAb, tezepelumab [23,104]. In late clinical development are also several biologics that block the eosinophil-promoting alarmin IL-33 or its receptor ST2 [105,106,107,108]. Apart from the biologics, dexpramipexole is an oral investigational drug that in recent clinical studies has been shown to robustly reduced both blood and tissue eosinophils, even though the mechanism of action remains to be identified [109].

Common to all eosinophil modifying drugs is uncertainties regarding what patient will respond. As a result, there is growing interest in discovering new biomarkers that more reliably predict response to eosinophil-targeted treatments. Potential avenues include transcriptomic and proteomic signatures of eosinophils isolated from blood or disease tissues, imaging-based methods to assess spatial distribution patterns, or integrative approaches that combine clinical, radiographic, and molecular data. Ultimately, a better understanding of eosinophil heterogeneity should enable more personalized treatment strategies and improve clinical outcomes.

## 6. Outstanding Questions on Eosinophil Effector Functions in Clinical Conditions

Despite the significant progress in eosinophil research and treatment of eosinophil-associated diseases, fundamental questions remain about the true scope of eosinophil functions in human disease. How do distinct eosinophil subsets modulate local immune responses in different disease phenotypes, or across anatomical compartments and patchy microenvironments within patients? Apart from type 2 inflammation, how do environmental factors such as microbiota [110], pollutants, or viral triggers shape eosinophil phenotypes? To what extent do human tissue eosinophils recapitulate the functions predicted by animal or in vitro studies?

Another key question relates to therapy response. Why do some patients with robust tissue eosinophilia fail to benefit from eosinophil-targeted treatments, while others experience dramatic improvements? Answers may reside in the heterogeneity of eosinophil states, differing microenvironmental cues, or the presence of concurrent inflammatory pathways. Indeed, the phenomenon of mixed type 2 and non-type phenotypes within individual patients has been observed in, for example, asthma and eosinophil-high COPD patients [14,61,111,112]. This phenomenon—a “mixed inflammatory phenotype”—which is likely present to a varying degree in most patients presenting with tissue eosinophilia, underscores the need to identify the local microenvironmental factors shaping eosinophil heterogeneity in real-life clinical settings.

Addressing the questions above will require carefully designed human studies, including tissue biopsies, advanced phenotyping techniques, and integrative analyses linking clinical data with cellular and molecular phenotypes. Examples of such initiatives are the 3TR, U-BIOPRED, and SARP consortia [113,114] in the asthma field and CEGIR in the field of eosinophilic gastrointestinal disorders (EGIDs) [115].

## 7. Novel Approaches to Decode Eosinophil Phenotypes in Diseased Human Tissues

The complexity of eosinophil biology demands high-resolution tools for context-based phenotyping. Single-cell techniques including scRNA-seq and high-end FACS approaches are powerful tools. However, eosinophils pose unique technical challenges. They are notoriously fragile and easily activated during purification [116], making in vitro functional assays and single-cell sequencing experiments prone to artifacts. Further, their high RNase content can degrade RNA, complicating transcriptomic analyses [117,118]. Techniques that avoid extensive cell manipulation, such as direct ex vivo isolation or immediate single-cell encapsulation [119], help to minimize activation artifacts.

An attractive approach, which avoids the eosinophil isolation issue and assesses cells directly in their microenvironmental tissue context, is histology-based spatial transcriptomic approaches. To this end, leading platforms like Visium and Xenium (10X Genomics, Pleasanton, CA, USA) and GeoMx^®^ (Nanostring, Seattle, WA, USA) allow customized or whole-genome transcriptomic data on tissue sections at a cellular resolution [120,121]. In practice, there are however challenges with robust cell delineation and analyzing spatially resolved large datasets. Nonetheless, rapid developments in spatial statistics, transcript-to-cell assignment strategies, and data interpretation [120,121,122] are driving significant progress in this field. Combining these methods with multiplexed immunohistochemistry histology-based mass spec techniques like MALDI variants and ICP-MS has the potential to further expose the true nature of eosinophil phenotypes across tissue niches and holistic cell patterns in real patient tissues.

As these technologies improve and become more accessible, the field will gain unprecedented insights into the real-time roles of eosinophils in human diseases. The goal is to not only catalog eosinophil subsets but also to understand how their functions evolve during disease progression, remission, and relapse, and how they contribute to or mitigate ongoing inflammation. Such knowledge could revolutionize the development of personalized therapies that modulate eosinophil activity in a context-dependent manner.

## 8. Summary and Future Perspectives

Eosinophils are surprisingly versatile immune cells whose behavior is shaped by local tissue signals, disease context, and interaction with other cell types. Rather than functioning solely as destructive granulocytes, they can also drive or regulate inflammation, promote repair, and actively coordinate immune responses. This complexity helps explain the highly varied clinical manifestations of eosinophilic disorders and the inconsistent responses to current therapies. Emerging single-cell and spatial phenotyping technologies, applied directly to patient tissues, offer the opportunity to resolve these spatiotemporal eosinophil states with greater precision. Such efforts could improve disease endotyping, guide targeted interventions, and potentially reveal new treatment strategies for eosinophil-associated diseases. Ultimately, harnessing eosinophils’ diverse roles—and tailoring therapies to specific eosinophil phenotypes—represents a promising frontier for personalized medicine.

## Data Availability

No new data were created or analyzed in this study.
